# Stressed at Work: Investigating the Relationship between Occupational Stress and Salivary Cortisol Fluctuations

**DOI:** 10.3390/ijerph191912311

**Published:** 2022-09-28

**Authors:** Thomas Gerding, Jun Wang

**Affiliations:** Division of Environmental and Industrial Hygiene, Department of Environmental and Public Health Sciences, College of Medicine, University of Cincinnati, 160 Panzeca Way, Cincinnati, OH 45267, USA

**Keywords:** occupational stress, workload, salivary cortisol, healthcare workers, stress diary

## Abstract

Chronic stress has been associated with a range of health disparities, but examination of occupational stress, especially in the wake of COVID-19, has been minimal for many careers. A novel methodology involving work stress diaries and collection of salivary cortisol was employed to determine correlations between occupations, occupational stressors, and how well these are related to the physiological response to stress exposure, the release of cortisol. While cortisol levels tended to follow typical circadian rhythm based on sampling times, cortisol levels also followed the subjective stress levels listed in the work stress diaries following linear regression analysis using the pooled study population data (*p* = 0.042). When comparing the stressors between the studied careers, participants who worked in the healthcare industry accounted for one-third of the total participants, but reported nearly half (42%) of the more severe occupational stressors listed in the diaries. Finally, the most commonly listed emotional reactions to exposures listed included feelings of stress, frustration, anger, anxiety, or overwhelm. As the workplace progresses from the pandemic, the opportunity to reduce occupational stress exposures in the workplace is at hand. Companies that work towards minimizing the stress faced by their workforce would have a healthier and more relaxed workforce.

## 1. Introduction

Work-related stress is a common occupational health concern and contributor to many aspects of health disparities, both in the United States and abroad [1,2,3,4,5,6]. Exposure to chronic stress has been related to a variety of possible disease outcomes including cardiovascular disease (CVD), hypertension, and poor mental health outcomes [7,8,9,10,11]. Occupational stress has been historically associated with interpersonal issues with co-workers or supervisors, poor work–life balance, poor job security, and overbearing workload stress [12]. This stress has also been exacerbated by the COVID-19 pandemic. Recent literature has found a majority of individuals experienced an increased workload due to the pandemic, and a majority also believed they could have potentially been exposed to COVID-19 in their workplace [13]. Further, certain career fields have been affected to different degrees regarding the pandemic. For example, while about two-thirds of the total participants from a recent study stated their workload increased since COVID-19, and 55% believed they could be exposed to the virus in the workplace, nearly 80% of healthcare workers stated concerns of workload and workplace exposure when stratified by industry [13].

Research on occupational stressors experienced by those working in abnormal settings has shown work-related stressors are associated with greater rates of depression, anxiety, and suicidality [14]. Further, during the COVID-19 pandemic, COVID-19-unit ICU nurses were more than 200% as likely to lack sufficient sleep and 300% as likely to be planning to leave their current department compared to their counterparts [15]. Little research has been performed on work-stress within certain sub-sectors of high-stress workforces, such as home healthcare workers (HHCWs), despite frequently experiencing a disproportionate share of exposure such as during COVID-19 and annual influenza outbreaks [16]. Finally, little time has been spent studying stressors of those who work in a home office setting [17]. Gaining knowledge on work-related stressors may be particularly beneficial to help towards understanding health disparities experienced by workers.

Workforces already vulnerable to excess stress faced additional stressors due to the pandemic. “Essential workers”, including home healthcare workers (HHCWs), worried about possible exposure in addition to their typical stressors. Although home healthcare is considered one of the fastest growing subsectors of healthcare and is more apt to encounter disproportionate shares of various exposures, it remains an understudied branch of healthcare [16,18,19]. Additionally, office workers, who have been an understudied group regarding occupational stress, faced occupational issues of their own as many were sent to work from home with nothing more than a laptop at their kitchen tables [17,20]. Most individuals surveyed also noted increased levels of tiredness and stress since working from home [20].

Measuring cortisol changes may provide insights into types of occupational stressors and how these may be minimized. As the body’s main stress hormone, cortisol is regulated by the hypothalamus–pituitary–adrenal (HPA) axis, which releases the hormone each day, following a circadian rhythm with levels typically highest in the morning and tapering off throughout the day [21,22,23,24,25]. However, additional cortisol may be produced due to external stress exposure to scenarios such as shift work, nature of the work, and organizational characteristics of work (i.e., high work demand or conflict with others), resulting in an increased heart rate and respiratory rate [26,27,28]. Chronically high cortisol levels can lead to altered immune system responses and digestive system suppression as cortisol stifles what the body considers non-essential functions during high stress scenarios [29]. Previous literature has found that serum unbound cortisol concentration is a more immediate measure of cortisol levels, as salivary cortisol concentration increases more rapidly as serum cortisol-binding globulin becomes saturated, although this delay was less than five minutes [30]. However, due to the simple and less invasive, salivary cortisol is still the recommended methodology within longitudinal, community-based studies as this has been found to be directly proportional to serum sampling and correlated well with adrenal function in tests of circadian variation [30,31]. As an individual is subjected to a variety of stressors throughout the day, these increases in cortisol levels could potentially be quantified, thereby correlating them to the stressor events. Upon quantification and characterization of psychological stress based on these biomarkers, it could be determined what scenarios may be deemed the most stressful in the workplace in conjunction with subjective exposures so employers would work towards minimizing the potential their occurrence through targeted prevention policies [32,33,34,35,36].

While the influence of occupational-related stress on the health of workers is increasingly being recognized [37,38,39,40,41,42], to date, relatively little research has been conducted with a focus on longitudinal salivary cortisol collection in conjunction with the documentation of daily stressor events. One study, by Eller et al. (2006), used survey response answers to describe participants, i.e., age, smoking status, children, and work-related questions, to name a few, but the lack of a diary limited the ability to truly connect cortisol fluctuations with what the participant was exposed to at a given time, on a given day [43]. In essence, this study was able to describe the degree of stress someone in a specific field experienced, but they were not able to explain what could have triggered that increase in cortisol to occur. Another study, focused on work stress in nursing, found that average cortisol levels on workdays were 60% higher than during off days and stress scores were lower based on completed surveys [44]. Here, again, though, potential reasons for the increases in cortisol levels were not studied during those working days. The ability to determine one’s cortisol levels following various stressor events could help to gain a greater understanding of what occupational scenarios quantitatively cause a greater level of stress in the individual. Furthermore, the COVID-19 pandemic highlighted how stress and external factors may negatively impact the mental well-being of the workforce. Although the world may be progressing out of the pandemic, mental health, i.e., stress and its causal factors, should clearly be given a similar level of importance as with the physical hazards present in the workplace to continually improve working conditions and to make sure employees are able to return home at the end of each day in the same condition in which they arrived.

The present study’s goal was to determine the degree of correlation between occupational stress and fluctuations in salivary cortisol, the most non-invasive approach to monitoring one’s cortisol levels, to physiologically characterize the impact of perceived stress experienced while working. No known study has been performed which used a work stress diary in addition to real-time saliva sampling by the participant in a working capacity instead of cortisol sampling within a clinical setting.

## 2. Materials and Methods

### 2.1. Sampling Strategy

Fifteen participants were recruited for this mixed nature, descriptive, and transversal study through convenience sampling via email and social media for this study, which was approved by the University of Cincinnati Institutional Review Board under protocol #2021-0681, ensuring the scientific design, methodology, study procedures, participant population, recruitment procedures, and consent processes all adhered to the code of ethics. Emails were concentrated on participants of a recent occupational stress study completed by the authors. Exclusion criteria included smoking status, lacking full-time employment, and diagnosis of Cushing’s Syndrome (a disorder affecting cortisol production). As individuals were recruited, each was given a work stress diary, sampling log, study protocol, and nine saliva sampling vials (Salimetrics, State College, PA, USA) in a cryostorage box (Salimetrics, State College, PA, USA). During three consecutive working days, participants were asked to document anything causing stress during working hours within the diary which included sections for occupation (i.e., nurse), date, time of day, intensity of stress on a 0–5 (none–highest) scale, stress duration, situation description, triggering event, if applicable, and any emotional behavioral reaction. They were also asked to describe whether the day would be considered typical of their job.

Concurrently, participants provided three saliva samples per day: once at the start of their shift, once at the start of the lunch break, and once at the end of their shift. It was initially planned to provide a stricter sampling schedule (i.e., 8:00, 12:00, and 4:00, for example), but was decided against to be as inclusive as possible to those who worked night shift or other schedules, such as three twelve-hour shifts or four ten-hour shifts per week. Participants were encouraged to use the small cryostorage box to keep samples organized and refrigerated within the refrigerator in their workplace, or home if they worked from home. Sampling logs were used to document the identification number of the sample vial and time/date when the sample was taken. Following the third sampling day, study materials were returned for enzyme-linked immunosorbent assay (ELISA) analysis. Participation in the study occurred from March through to April of 2022. Preliminary power calculations found that a total of 135 samples (fifteen participants providing three samples per day) was sufficient in conjunction with an α error probability of 0.05 and a power of 0.80 towards the completion of a two-way ANOVA.

### 2.2. Salivary Cortisol Analysis

As the participants returned the study supplies, all saliva samples were stored in a freezer set at −20 °C until there were four participants’ samples which would fill the 96-well plate in addition to the necessary standards, controls, and blanks. No participant’s samples were stored in a freezer for longer than one week although they may be frozen for up to six months according to the assay kit’s manufacturer instructions (Salivary Cortisol Enzyme Immunoassay Kit, Salimetrics, State College, PA, USA). When enough samples were collected to warrant an analysis, all reagents were brought to room temperature and all samples were thawed completely, vortexed, and centrifuged at 1500× *g* for 15 min. Two of the wells in the microplate were replaced with non-specific binding wells and 25 µL of standards, controls, saliva samples, and assay diluent were pipetted into various wells as appropriate. All wells were run in duplicate. A 1:1600 enzyme conjugate was diluted by adding 15 µL of the conjugate to 24 mL of assay diluent and 200 µL of this solution was added to each well using a multichannel pipette.

The contents of each well were then mixed using a plate rotator for five min at 500 rpm and was then incubated at room temperature for one hour. After the hour concluded, the plate was then washed four times with a wash buffer solution by pipetting 300 µL of the wash buffer into each well and blotting the plate with a paper towel prior to up righting the plate between each wash. Next, 200 µL of tetramethylbenzidine (TMB) substrate solution was added to each well and the plate was again mixed on a plate rotator for an additional five minutes at 500 rpm. The plate was then incubated at room temperature for an additional 25 min at which point 50 µL of a stop solution was added to each well. Finally, the plate rotator was used for an additional three minutes at 500 rpm, the bottom of the plate was wiped dry, and the plate was entered into a plate reader at 450 nm.

All samples were analyzed for salivary cortisol in accordance with the ELISA kit manufacturer’s instructions (Salivary cortisol enzyme immunoassay kit, Salimetrics, State College, PA, USA). After raw data collection from ELISA tests, the following calculations were used to determine cortisol levels from each sample. Average optical density (OD) was calculated for duplicate wells. The average OD for the non-specific binding wells were subtracted from the average OD of all other wells. The percent bound (B/B_0_) was then calculated for each standard, control, and saliva sample by dividing the average OD of each well by the average OD of the zero wells. Finally, the µg/dL concentration of each standard and the resulting B/B_0_ for each were used to construct a 4-parameter non-linear regression curve fit. Once completed, the averaged B/B_0_ value for each sample was applied to the equation provided from this model which facilitated cortisol value determinations.

### 2.3. Statistical Analysis

Two-way ANOVA was completed to determine correlations between the time of day when a sample was taken versus the cortisol concentration, and linear regression was performed to determine correlations between the cortisol level of a given sample with the self-reported stress level of the nearest preceding stressor event logged in the diary. Regression models were first constructed for each participant, then pooled together to include all data in a single regression. Time of day was segregated into three categories, first, second, and third sample, which would have equated to morning, midday, and afternoon for many of the participants. Levels of statistical significance were set at *p* ≤ 0.05. Statistical analyses were performed within SigmaPlot 14.0 (Inpixon, Palo Alto, CA, USA). Lastly, a qualitative analysis and summary of stressor events and subjective stress levels was completed.

## 3. Results

### 3.1. Study Demographics

Participants ranged from numerous careers including: R&D scientist for a multinational consumer goods corporation, work-from-home (WFH) supervisor for a Fortune 500 health insurance company, WFH college admissions advisor, marketing, WFH project manager for luxury vacation rentals, WFH internet marketer, WFH information security manager for a healthcare system, a master’s level graduate student, weight loss specialist/health center manager, wastewater treatment biologist, accounting specialist, and four nurses. Of the fifteen, nurses made up 26.7% of the population, those in a WFH capacity made up 33.3%, and the rest accounted for 6.6% each (i.e., one graduate student, one biologist, etc.). Of the nurses, one was a respiratory therapist, and another worked partly in a HHCW capacity. Four participants were men (26.7%), eleven were women (73.3%), and ages ranged from twenty-eight to sixty-two. All participants worked day shifts typically of either five eight-hour shifts, four ten-hour shifts, or three twelve-hour shifts except for the respiratory therapist who worked three twelve-hour overnight shifts per week.

### 3.2. Correlation between Sampling Time and Cortisol Level

The two-way ANOVA results found the difference in mean values among the levels of salivary cortisol from study subjects was greater than would have been expected by chance regarding effects from the time of shift when a sample was taken (*p* < 0.001, study subject, F = 11.462). Here, although some increased during the day, cortisol tended to wane throughout the working day, following typical circadian rhythm. Sampling error for the dataset with a confidence level at 0.05 was found to be 1.387. As not all participants worked identical shifts based on time of day, sampling time was defined as sample one, two, and three, during each shift (time of day, F = 3.642, *p* < 0.030). So, while not all samples were taken at the same time of day between participants, they were statistically different from one another when comparing the samples based on the order collected.

### 3.3. Correlation between Cortisol Level and Self-Reported Stressor

The linear regression analysis tested the relationship between self-reported levels of stress occurring most closely to the nearest sample provided. Only two individuals’ regression models were found to be significant in addition to the pooled model. The pooled model contained all 135 samples and was found statistically significant (*p* = 0.042). The constant variance test, Spearman rank correlation, passed and had a power of 0.53, but had an R-squared value of only 0.0308.

### 3.4. Differences within Shift

**Figure 1:**
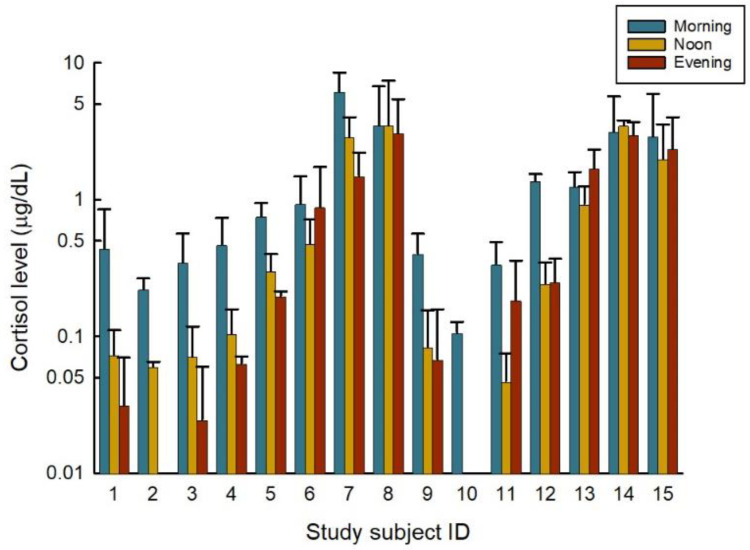
Log-transformed graph of interpersonal differences between sampling times.

Most samples followed a trend of highest in the morning and tapering downward during the day, similar to typical circadian rhythm. Figure 1 compares cortisol levels and participant, separated by the daily sample average for first, second, and third daily samples. The second participant’s third daily mean and the tenth’s participant’s second and third daily mean concentration were all below 0.01 µg/dL on the graph as they were nearly a concentration of 0 µg/dL. Participants six, eleven, twelve, thirteen, fourteen, and fifteen showed increased average concentrations of the final daily samples in their sampling period compared to their second daily samples and sometimes even their first daily samples.

### 3.5. Stress Diary Information

One of the two participants with statistically significant correlations between the self-reported stress levels and their corresponding cortisol levels included a WFH information security manager for a healthcare system. This person experienced self-reported stress levels ranging from 1 to 3 out of 5 on day one and levels of either 1 or 2 on the second and third day of the study period. The stressors given a level of 3 involved unexpected spreadsheet formatting changes in a collaborative file and running late for a dentist appointment due to a work meeting running over schedule. The lesser occupational stressors predominantly dealt with prepping for the day at hand, a slight overburden involving work tasks (i.e., ticket queues, unread emails, and review processes), and stress over homelife related factors as this individual worked from home.

The second participant with statistically significant correlations between their self-reported stress levels experienced and their cortisol levels was a registered nurse who worked partially in a HHCW capacity. This participant noted higher levels of stress over the course of their three-day participation overall ranging from 2–4 out of 5. On day one of the participants study period, the individual noted a stress level of 4 when preparing for the day as they had accidentally slept a little too long. Closer to noon, this individual then noted a stress level of 3 due to a home patient’s family needing their visit moved so the participant was stressed over juggling patient times. On the second day of the study period, the nurse expressed stress levels of 3 and 2 due to others working from home so many calls came to her, interrupting her work, and bad traffic, respectively. Finally, the third day of the study period saw the participant experience a stress level of 2 when getting prepared for a clinic followed by a stress level of 4 in the afternoon when a patient passed away very unexpectedly, and she experienced guilt from this.

The Supplemental File, Table S1, outlines and summarizes any of the occupational stressors ranked three or higher experienced by the fifteen participants over the course of the study period. Many of these stressors dealt with keeping up with work tasks and an overburdened workload/schedule. When focused on the stressors given a three or higher, of the 59 total stressors listed, 25 of these were from participants who worked in healthcare, either the four nurses or the weight loss specialist. Of the 15 participants, everyone experienced at least one occupational stressor level three or higher over the course of their three-day study period. The two individuals who experienced the fewest higher stressors included the internet marketer who had experienced one level three event involving an unforeseen problem at work and one of the nurses who had experienced one level three event due to lab results coming back wrong which made her anxious. On the other hand, the individual who experienced the most amount of higher-level stressor events was one of the other nurses who reported experiencing three level threes, three level fours, and three level fives within the three-day period. Nearly all of these stressor events involved understaffing issues at the hospital such as a lack of help for the patients, being overburdened with tasks, and caring for sick patients.

Many of the diary entries reported the study days were typical, but a few had abnormalities during their day. The accounting specialist was newer to her job and was learning how to complete payroll for employees and navigate a new reporting system. The project manager had to work on what was typically one of her days off. The person in marketing had a team meeting and an evening activity with work. One of the nurses worked in a home healthcare capacity during one of her three study days although this was fairly regular. Finally, the health center office manager dealt with a missing employee during the second day of the study period to the point where police needed to be involved.

Table 1 shows the frequencies with which various emotional descriptors were written within the emotional behavioral reaction column of each diary entry. Although a total of 128 stressor events were inscribed within all fifteen participants’ diaries, the emotional behavioral reaction column was not completed for all entries and a total of 121 emotional reactions were recorded. With 16 uses of “stressed” or “stress”, this appeared to be the most popular emotional behavioral reaction provided at 13.22%. Closely behind “Stressed” came various feelings of upset at work situations the participants experienced including: “Frustrated” at 12.40%, “Angry/Mad/Pissed” at 11.57%, “Anxious/Unease/Restless” at 10.74%, and “Overwhelmed/Pressured” at 8.26%. All diary entries related to the aforementioned frequent behavioral reactions dealt with feelings due to situations at work, such as an overbearing workload, but no conflicts with co-workers or supervisors were noted associated with any of these emotions. In that regard, examples of emotional reactions which dealt with co-workers or supervisors included being frustrated and mad over writing up an employee due to a policy that the participant did not believe in, worry, anxiety, and stress over having to replace an employee who received a promotion, and nervous/overwhelmed over firing an employee. Of the emotional reactions written least frequently, the following all had an occurrence of two each: “Shame/Guilt”, “Confused/Surprised”, “Tired”, “Frazzled”, “Dread/Fear”, and “Impatient”.

## 4. Discussion

### 4.1. Correlation between Sampling Time and Cortisol Level

Following the data analysis of all fifteen participants after the conclusion of the ELISA analyses, it was determined there was a statistically significant relationship between a given cortisol level and the time of day it was taken utilizing the two-way ANOVA. This trend was to be expected as cortisol levels tend to follow a circadian rhythm with the highest levels typically occurring upon waking each day [21,45]. Further, the importance of documenting the time of sampling towards sound statistical analysis has been outlined in previous literature, due to fluctuations over the course of the day [46]. Time of day was segregated into three categories: first sample, second sample, and third sample. For most participants, this would have equated to the morning, midday, and afternoon sample. However, the rapid response team nurse worked overnight shifts, but her cortisol levels followed the same trend as the analysis presented with levels declining from first to third sample per day, although she worked from the evening into the morning.

### 4.2. Correlation between Cortisol Level and Self-Reported Stressor

Of the fifteen participants included in this study, only two individuals’ datasets were deemed statistically significant regarding their relationship between stress levels and corresponding cortisol levels. However, when pooling the data from the entire study population, this was determined to be statistically significant, demonstrating there was a notable relationship between one’s subjective stress level assigned to an occupational stressor event and their physiological response of cortisol release. Unfortunately, the R-squared value with this relationship was only 0.031. Nevertheless, while the model presents the idea only 3.1% of the variation is accounted for, there could have been a variety of confounders contributing to one’s cortisol levels. Stressors not accounted for in a participant’s diary, such as occupational stressors a participant may not have deemed of adequate magnitude to include in the diary, personal/familial stressors which one may not have wanted to include, or simply forgetting to document a stressor, could have been somewhat responsible for the poor predictive value provided. Additional confounders could include aspects such as smoking habits, time of awakening, alcohol consumption, body mass index, and medication, of which smoking and alcohol consumption were both controlled for through exclusion criteria and participant instructions [47,48,49,50,51].

Additionally, if the only point of the linear regression model was prediction, the model would indeed poorly predict one’s cortisol level following an occupational stressor. Yet, the purpose was to determine if there was a small, but reliable relationship between the two factors, which was the case. Admittedly, the ability to potentially reduce one’s occupational stress exposure by 3% through minimizing the exposures noted in the diaries would not seem to legitimately reduce one’s cortisol levels, sampling a larger participant pool would potentially increase the statistical validity of the present study, ideally improving the associated R-squared and power level. Another tactic to improve the validity may be to simply increase the longevity of the study period compared to three consecutive days for each participant if a smaller study population is used. This holds true to previous literature which found seemingly poor covariance between perceived stress and salivary cortisol to be typical due to the complex interplay of the neurobiological events linking perceived stress with HPA axis activation [30]. The ability to collect total cortisol in blood, salivary cortisol, and adrenocorticotropic hormone levels would paint the clearest picture of cortisol levels within the individual, although impractical in a community-based study where multiple samples per day are necessary. For this reason, the recommended methodology would still be the sampling of saliva following a standard cadence [30,52].

### 4.3. Temporal Difference

While nearly two-thirds of the participants cortisol trends tended to follow the typical pattern of being greatest in the morning and declining throughout the day, there were a handful who tended to have greater cortisol levels in their third sample each day. This would infer something stressful may have been experienced by each of these individuals. Upon consulting the work diaries submitted for each of the participants who had these trends, participants 6, 11, 12, 13, 14, and 15, experienced atypical stress in their work shift during their sampling days. Participant 6, for example, worked from home while caring for their toddler. In the healthcare realm, participant 11 was the health center manager whose employee went missing, participant 12 was a nurse who found out one of her patients had passed away, and participant 13 appeared to be in a short-staffed work setting based on many of her diary entries. Participant 14, the wastewater biologist, expressed feelings of frustration towards the afternoon one day when a supervisor left work early and expected him to fix an unplanned outage. Finally, participant 15 was recently hired in an accounting capacity and was learning how to process payroll during the week of the study. Fortunately, none of the participants worked irregular shift work (i.e., alternating work schedules) which has been found to increase cortisol excretion upon waking in the morning and a slower regression over the course of the working day [45].

### 4.4. Stress Diary Information

Regarding the more intense stressors experienced by the participants, nearly half of the 59 events which were described as a stress level of 3, 4, or 5 out of 5 were experienced by those in healthcare, demonstrating the trend in healthcare occupations typically consisting of a great level of stress. Further, three of the seven level-five stressors were experienced by one individual in healthcare. What might be considered a silver lining, nearly all these greater stressors experienced by the healthcare participants involved stress over providing care for their patients as opposed to stress involving their superior or workplace conflict with co-workers. In fact, the only events logged in the diaries of the healthcare participants that involved co-workers involved a couple working from home and one who went missing. This individual was eventually located by authorities, but then terminated their employment within the same week.

Although all participants experienced a stressor event they described as at least a 3 out of 5, it was natural for all participants to experience varying amounts of stressor events and various degrees of stress during the three working day period they had participated in the study. This was largely dependent on if the study period were considered typical working days for the individual or if abnormal stressors were experienced. For example, one of the nurses had a typical week according to her diary and listed a total of seven stressor events, with only one level three event. On the other hand, while the wastewater biologist listed only four stressor events over the course of the three days, two were level four and one was level three, appearing that his days were more sporadically stressful although he listed each day as typical. Worse yet, although one of the nurses listed her days as typical, she also documented nine stressor events level three or greater (3 = 3, 4 = 3, 5 = 3) which gives her job the appearance that she is typically very stressed even on what would be considered a normal day.

Lastly, the 121 emotional behavioral reactions given for any of the diary entries were quantified into a frequency table. The most frequent emotional behavioral reactions documented included: “Stressed” at 13.22%, “Frustrated” at 12.40%, “Angry/Mad/Pissed” at 11.57%, “Anxious/Unease/Restless” at 10.74%, and “Overwhelmed/Pressured” at 8.26%. Many of the associated stressor events with these emotional reactions involved issues with their work or their job, but fortunately nearly no issues were noted involving workplace conflict with others. Common issues involve stress over one’s job typically consisted of feeling overwhelmed with a workload, being short-staffed, or poor opinions of workplace policies.

### 4.5. Limitations

The participants were recruited using convenience sampling in a large, midwestern metropolitan area up until fifteen individuals were recruited. For this reason, the findings of this study may not completely reflect occupational stressors experienced by workers in general, but does provide insight regarding common stressors in the workplace. For example, the qualitative results showed healthcare appeared to be a commonly stressed career field.

Second, the work stress diary entries were based on the perceptions of the individuals, especially the intensity of the stress experienced. Subjective perceptions about the level of stress experienced, if the day was typical, etc. have the potential be biased as what one may consider a stressor event of 5, someone else may describe the same event as a 3. Further, there may have been events that occurred which a participant deemed not appropriate to enter into the work diary (i.e., if they felt the need to concentrate specifically on occupational stressors, but something familial caused them stress at work, or if someone had a physiological stress response, but did not think the event severe enough to log the occurrence). For this reason, bias may exist to the extent of participants underestimating the breadth or depth of stressors experienced while working. Future work could aim to be carried out following the end of the pandemic and investigate how well one’s subjective sense of severity of stress correlates with physiological responses, measuring salivary cortisol as well as salivary alpha-amylase, an enzyme whose presence has been found to increase following exposure to external stress [45].

Finally, due to budgetary constraints and the goal of methodology validation, only fifteen participants were recruited for this study and only a total of nine saliva samples were collected over the course of three consecutive working days. Future work hopes to collect for a longer study period using a larger population pool which would potentially increase the statistical validity of the study and improve the foundation of knowledge. Ideally, a greater depth and breadth of knowledge will lead towards the prevention of illnesses associated with chronic stress so as to reduce workplace absenteeism or economic repercussions [53]. Further, we hope to collect data on alpha-amylase fluctuations in addition to changes in salivary cortisol levels. Studying targeted industries, such as home healthcare workers or those that work from home may also help to understand he stressors experienced by growing work sectors.

## 5. Conclusions

The results from this study illustrate a variety of common stressors experienced by a variety of workers: healthcare, WFH, information technology, scientists, and a few other white-collar workers. Levels of salivary cortisol levels tended to follow typical trends due to circadian rhythm although there were some individuals who experienced increased cortisol levels towards the end of their work shift, potentially due to exposure to occupational stress. Although the correlation between cortisol levels and the levels of self-reported stress occurring most closely to respective sampling times was determined to be statistically significant, the low R-squared value could have been due to the various confounders associated with stress. Expanding the participant pool and the sampling period could aid in allocating for greater validity. Qualitatively, it was shown that although participants who worked in healthcare accounted for one-third of all participants, they documented 25 of the 59 intense stressors reported. Based on the emotional behavioral reaction frequencies, it was common for participants to feel emotions involving stress, frustration, anger, anxiety, and being overwhelmed. In addition to minimizing the risk of occupational illness or injury, workplaces should allocate greater resources for mental health awareness and intervention. Besides a reduction in the triggers which may spike one’s cortisol levels, minimizing occupational stress could also improve workplace retention, productivity, and the interpersonal relationships between co-workers.

## Figures and Tables

**Table 1:** Emotional behavioral reaction frequencies in the occupational stress diaries.

Emotional Behavioral Reaction	Count	Percent
Stressed	16	13.22%
Frustrated	15	12.40%
Angry/Mad/Pissed	14	11.57%
Anxious/Unease/Restless	13	10.74%
Overwhelmed/Pressured	10	8.26%
Rushed	9	7.44%
Annoyed/Aggravated/Irritated	9	7.44%
Worry	8	6.61%
Nervous/Agitated	8	6.61%
Disappointed/Bummed/Sad	4	3.31%
Concerned/Distressed	3	2.48%
Shame/Guilt	2	1.65%
Confused/Surprised	2	1.65%
Tired	2	1.65%
Frazzled	2	1.65%
Dread/Fear	2	1.65%
Impatient	2	1.65%
Total	121	100.00%

## Data Availability

The data that support the findings of this study are openly available in Figshare. ELISA Plate IDs. Available online: https://doi.org/10.6084/m9.figshare.20173208.v1 (accessed on 27 September 2022).

## Supplementary Materials

The following supporting information can be downloaded at: https://www.mdpi.com/article/10.3390/ijerph191912311/s1, Table S1: Summary of the intense occupational stressors experienced by participants (intensity of stress = 3, 4, or 5), can be accessed on the IJERPH website.
