# Supplementary material for: Stressed at Work: Investigating the Relationship between Occupational Stress and Salivary Cortisol Fluctuations
Source: Int J Environ Res Public Health. 2022 Sep 28;19(19):12311. doi: 10.3390/ijerph191912311 (PMC9564551; doi:10.3390/ijerph191912311)
Supplement: Supplementary file 1 [file ijerph-19-12311-s001.zip › ijerph-1926539-supplementary.pdf]

Table S1. Summary of the intense occupational stressors experienced by participants (intensity of stress = 3, 4, or 5).

Participant's Career	Stress Level	Stressor Entry	Was this a typical day?
Principal Scientist	3	Positive stress of having many urgent things to do and limited time.	Yes
Principal Scientist	3	Positive stress. Exciting discussions & experiment planning.	Yes
Health Insurance Supervisor	3	Found out one of my associates made a big mistake and I have to write her up. But I don't think the reasoning for this policy is fair. So I don't agree w/error. Frustrated.	Yes, typical Wednesday full of meetings from 8-430pm
Health Insurance Supervisor	3	Meeting with manager about my team lead's poor communication skills. Felt stressed and overwhelmed.	Yes, a normal Thursday working from home. Usual amount of stress.
Health Insurance Supervisor	5	I had to fire one of my associates today at 3pm. At 11am I started to prepare. Nervous, overwhelmed	Yes, a normal Thursday working from home. Usual amount of stress.
Online University Admission Advisor	3	Issues w/tech – not able to have access. I feel angry at this situation.	Yes
Online University Admission Advisor	4	Continued tech issues – dog bothering me. Anger at dog, annoyed about work	Yes
Online University Admission Advisor	3	Tech issues resolved – but now dealing and trying to catch up on work. Overwhelmed	Yes
Online University Admission Advisor	3	Given reports to complete/audit I didn't know about. Frustrated	Yes
Online University Admission Advisor	3	Struggling with work-mail merge – long process – needs to be completed tomorrow. angry	Yes
Online University Admission Advisor	3	Stress about work again – about to start sorting things out – getting emails about other things. Worried & overwhelmed	Yes
Online University Admission Advisor	4	Working on project – sending lots of emails – very demanding. Stressed	Yes
Marketing	3	At work, couldn't compute a task for my leader's leader b/c I didn't have the tools necessary. Anxious, worried	No, team meeting + evening activity
Marketing	3	Team building exercise to build the tallest Lego tower w/in 30 min. w/o it falling. Friendly competition.	No, team meeting + evening activity
Marketing	3	Rushing to get work done to get to a family event I'm not looking forward to. Stressed, angry, rushed	No, team meeting + evening activity
Marketing	3	Realized I missed my mid-shift sample	Yes
Project Manager	4	Difficult chat w/co-worker. Stressed	Yes, work day, but long DR apt in middle
Project Manager	3	Last minute important request. Confused	Yes, work day, but long DR apt in middle
Project Manager	3	Going over large to-do list w/co-worker. Annoyed	Yes
Project Manager	3	Co-worker forgetting important request. Annoyed/stressed	No, I don't usually work today
Internet Marketer	3	Unforeseen problem at work.	Yes
Information Security Manager	3	Excel formula formatting date. DATEVALUE function needed	Yes
Information Security Manager	3	Dentist appt traffic, late. Next appt. July. Work meeting ran over.	Yes
Graduate Student	3	Too many assignments to do	Start of the work week
Graduate Student	5	Rework on lab reports	Yes
Graduate Student	5	Working with the reports. Stressful	Yes
Graduate Student	5	Working with the reports. Stressful	Yes
Graduate Student	3	Finally done but worried with others. Relief but worried.	Yes
Nurse	3	Lab results done wrong. Anxious	Yes

Rapid Response Team Nurse	4	Labs weren’t as good as expected. Frustrated nothing was working	Yes
Rapid Response Team Nurse	3	Getting an arterial blood gas stick on a dehydrated pt. Rushed, stressed	Yes
Rapid Response Team Nurse	3	Last minute bed change due to a shit explosion (literally). Had a lot to do, don’t like the smell of C. diff.	Yes
Rapid Response Team Nurse	3	Patient’s whole family in room – very sick patient. Sad, empathy	Yes
Rapid Response Team Nurse	3	Transporting my ventilated pt. To MRI. MD insisted on transporting very sick pt against my advice. Stress, uneasy	Yes
Rapid Response Team Nurse	4	Setting up nitric on COVID pt. Rushed	Yes
Health Center Office Manager	4	Employee not at work and no one heard from them in 7 days. Worried.	No, missing employee
Health Center Office Manager	4	Police notified of missing employee. Unknown emotional distress – felt sick to stomach	No, missing employee
Health Center Office Manager	3.5	Appts canceled & boss asking about current policies. Boss wanting to implement new cancellation policies.	No, missing employee
Health Center Office Manager	3	Having work load doubled & having to take clients that I wasn’t prepared for. Anxiety & overwhelmed at situation.	No, missing employee
Health Center Office Manager	3.5	Client arriving late. When client is late, it makes my schedule run behind.	Yes
Registered Nurse	4	I have to get up earlier than usual on Wed. I’m not a morning person.	Yes, sees home care pts. Instead of clinic
Registered Nurse	3	A family changed visit time. Stressed at having to move pt. Visit.	Yes, sees home care pts. Instead of clinic
Registered Nurse	3	2 genetic counselors on our team working from home. All calls coming to me. Can’t get other work done.	Yes
Registered Nurse	4	Patient died very unexpectedly. Sad – wondering if I could have done anything differently.	Yes
Wastewater biologist	3	Training new staff. Equipment failures. Busy & high stress afternoon	Yes
Wastewater biologist	4	Boss made unnecessary changes to process and took vital equipment offline; All because they didn’t follow through on scheduling ahead. Anger	Yes
Wastewater biologist	4	Above mentioned supervisor left early from work w/o resolving issues or leaving any instructions. Frustration	Yes
Accounting Specialist	3	Trying to start payroll batch on my own. Feeling frazzled trying to remember the step by step	Payroll day, we process payroll for the entire company
Accounting Specialist	3	My desk is full of paperwork and it’s approaching the end of the day. It’s been busy! I’m moving quickly.	Payroll day, we process payroll for the entire company
Accounting Specialist	3	In a training call for new ERP system watching co-worker navigate. I was doing a similar training before.	No, training in new ERP system.
Registered Nurse	5	Getting report on 5 patients for the day. Sick challenging patients. Worried.	Yes
Registered Nurse	5	Patient needing to use commode but RN stuck in another room with sick pt. Patient messed his pants. Family & pt. were upset. Frustrated & angry.	Yes
Registered Nurse	5	Rapid response – new nurses pt. In midst of stroke. Helping him through. Fear, worried, relief, adrenaline rush!	Yes
Registered Nurse	3	Catching up on everything. Behind because of rapid response.	Yes
Registered Nurse	4	Start of shift – a charge nurse with full team of sick pts. No help...	Yes
Registered Nurse	4	Busy sending two patients home at same time and getting everything together. Rushed	Yes

Registered Nurse	4	New admits almost as soon as beds are cleaned from old ones. Still not caught up on computer charting. No unit clerk, no pch. Trying to make pts. feel they are getting the care they deserve.	Yes
Registered Nurse	3	I scheduled appt. But know they are short sort of on call wondering if I’m needed. Anxiety.	Yes
Registered Nurse	3	Had to go into work 6 hours. Sitting for dementia patients. Not a bad afternoon	Yes
